# The Time of Prenatal Androgen Exposure Affects Development of Polycystic Ovary Syndrome-Like Phenotype in Adulthood in Female Rats

**DOI:** 10.5812/ijem.16502

**Published:** 2014-04-01

**Authors:** Fahimeh Ramezani Tehrani, Mahsa Noroozzadeh, Saleh Zahediasl, Abbas Piryaei, Somayeh Hashemi, Fereidoun Azizi

**Affiliations:** 1 Reproductive Endocrinology Research Center, Research Institute for Endocrine Sciences, Shahid Beheshti University of Medical Sciences, Tehran, IR Iran; 2 Endocrine Physiology Research Center, Research Institute for Endocrine Sciences, Shahid Beheshti University of Medical Sciences, Tehran, IR Iran; 3 Department of Biology and Anatomical Sciences, Faculty of Medicine, Shahid Beheshti University of Medical Sciences, Tehran, IR Iran; 4 Endocrine Research Center, Research Institute for Endocrine Sciences, Shahid Beheshti University of Medical Sciences, Tehran, IR Iran

**Keywords:** Androgens, Exposure Time, Fetus, Polycystic Ovary Syndrome, Rats

## Abstract

**Background:**

Polycystic ovary syndrome (PCOS) is one of the most common reproductive disorders in women. Previous studies have shown that prenatal exposure of female fetuses to androgen can be considered an important factor in the development of PCOS.

**Objectives:**

In the present study we aimed to examine the effects of prenatal exposure of female rat fetuses to previously documented doses of testosterone on different embryonic days on the development of PCOS phenotype in adulthood.

**Materials and Methods:**

Pregnant rats were divided into four groups, experimental and control groups. Three mg of free testosterone was administered subcutaneously to experimental group 1 on gestational days 16-19, daily and 20 mg on day 20, to experimental group 2, and the controls received solvent at the same times. Female offspring of these mothers aged between 90-100 days were examined for development and function of the reproductive system. Independent-sample student t test was used to compare the results between the experimental groups and controls.

**Results:**

Anogenital distance (P < 0.001) and clitoris length were significantly increased in the offspring of both experimental groups (P < 0.001 and P < 0.05 respectively). Nipples were not formed in the offspring of experimental group 1, whereas in experimental group 2 the number of nipples was unchanged. Vaginal length was significantly decreased in the offspring of experimental group 1 (P < 0.001), whereas in experimental group 2, no significant difference was observed. In the offspring of experimental group 1, hormonal profiles did not differ, but in experimental group 2, levels of testosterone (P < 0.05) and LH (P < 0.01) were significantly increased, but estrogen (P < 0.05) and anti-Mullerian hormone levels (P < 0.001) were significantly decreased. A significant increase in the number of preantral and antral follicles was observed in the ovaries of offspring of experimental group 1 (P < 0.05); whereas there was no such a difference in experimental group 2.

**Conclusions:**

The time of prenatal exposure to androgens may have a significant role in the development of PCOS. Increased prenatal androgen levels are associated with hormonal changes and morphological disorders of the reproductive system. Therefore, avoiding exposure to androgen excess during critical periods of fetal development may prevent or reduce adulthood PCOS manifestations caused by prenatal excess androgen.

## 1. Background

Polycystic ovary syndrome (PCOS) is one of the most common reproductive disorders in women with prevalences of 8-12% in reproductive ages (1). The most important reproductive and metabolic characteristics of PCOS are oligomenorrhea, amenorrhea, oligoovulation, anovulation, infertility, luteinizing hormone (LH) hypersecretion, hyperandrogenism and hyperinsulinemia (2, 3). Due to its peripubertal onset and familial clustering, PCOS is known as an autosomal dominant genetic disease (4). On the other hand, there is a lack of reliable association between genotype and phenotype of this syndrome (5), indicating that beside genetic factors, environmental factors may also contribute to development and appearance of PCOS. 

Previous studies have shown that prenatal exposure of female fetuses to androgen hormones can be considered an important factor in the development of PCOS (6); such exposure also induces changes in female reproductive phenotype in adulthood (7). Other studies conducted in humans and animals suggest that exposure of females to androgen during early life (prenatal, perinatal or early postnatal life) may lead to development of PCOS phenotype in adulthood (2, 8, 9). Both congenital adrenal hyperplasia caused by 21-hydroxylase deficiency and congenital excess androgen secretion from the adrenal can lead to an increased incidence of PCOS (2). These studies have shown that androgen excess during the fetal period may be associated with PCOS. Recently, experiments in animals revealed that the etiology of PCOS may be related to excess androgen, which could interfere with the function of the hypothalamus-pituitary-gonad axis and contribute to reproductive dysfunction in adults (2). Several studies have introduced animal models of PCOS to assess the effects of prenatal exposure of animals to androgen excess; for example in mice, rats, sheep and monkeys, prenatal androgenized animals showed many symptoms of PCOS after puberty such as hyperandrogenemia, increased LH secretion, polycystic ovary, oligoovulation and some others (2, 5, 10, 11). Rat or mouse models of PCOS may be preferred to other animal models, due to their stable genetic backgrounds, ease of handling and maintenance, shorter reproductive lifespan and generation times, feasibility of genetic manipulations and their short lifespan. It has been reported that the effect of androgen on the female reproductive system is highly influenced by the time of exposure (2, 5). 

## 2. Objectives

We aimed to examine the effects of prenatal exposure of female rat fetuses to previous documented doses of testosterone on different embryonic days on the development of PCOS phenotype in adulthood.

## 3. Materials and Methods

### 3.1. Animals

Thirty-two female Wistar rats with body weights of 170-190 g (age 70-90 days) were obtained from the animal center of Research Institute for Endocrine Sciences (RIES) of Shahid Beheshti University of Medical Sciences (SBUMS) (Tehran, Iran). Male and female animals (one each) were housed in a polypropylene cage (43 cm×30 cm×15 cm) overnight in an environmentally controlled room (temperature 22 ± 3°C, relative humidity 45-55% with 12-h light/dark cycles). Observation of the vaginal plug was considered as the first day of pregnancy. Pregnant rats were randomly divided into two experimental groups and two vehicle groups (controls), (8 in each group). Animals were handled according to the principles of laboratory animals care approved by the local ethics committee of Research Institute for Endocrine Sciences (RIES) of Shahid Beheshti University of Medical Sciences (SBUMS) (Tehran, Iran); (320 EC 90.09.07). 

### 3.2. Hormone Injection

The time and dosage of testosterone injection were set according to previous studies (2, 12). 

In experimental group 1, pregnant rats were injected daily with 3 mg of free testosterone subcutaneously (T1500, Sigma, Germany) dissolved in a 500 µL cocktail containing sesame oil (S3547, Sigma, Germany) and benzyl benzoate (B6630, Sigma, Germany) with a ratio of 4:1, on gestational days 16-19. Each rat in control group 1 was injected with 500 µL of the cocktail. In experimental group 2, pregnant rats were injected with 20 mg of free testosterone subcutaneously dissolved in 1 ml solvent on day 20 of the gestational period, while its control group received 1 ml of the solvent. 

The female offspring of these mothers were examined for the development and function of the reproductive system at the age of 90-100 days (in adulthood) and for anogenital distance (AGD); (which is the distance (mm) between the cranial edge of the anus and the base of clitoris) at days 6, 30 and 60.

### 3.3. Determination of Body Weight, Morphological Parameters and Organs Weight

Body weight of female offspring was measured at birth and days 15, 30, 45 and 60 after birth. AGD was measured (mm) by Vernier calipers at the age of 6, 30, 60 days. In adulthood all morphological parameters including AGD, clitoris length, number of nipples, vaginal length and kidney to ovary (k-o) distance were measured; prenatal androgenized female offspring were also examined for existence of male organs including ventral prostate, seminal vesicle and bulbourethral glands. Body weight and the weights of brain, adrenal glands and ovaries were determined. Absolute weight of organs was measured by digital scale (Sartorius, Germany) with 0.1 mg accuracy.

### 3.4. Blood Sampling

Female offspring were anesthetized by intraperitoneal (i.p) injection of pentobarbital sodium (P3761, 5mg, Sigma, USA) dissolved in normal saline 0.9% (60 mg/kg body weight) in adulthood. Blood was collected from the abdominal aorta in micro centrifuge tubes, centrifuged at 10000 rpm, 4˚ C, for 5 min. Sera were stored at -80˚C for subsequent measurement of hormone levels (Testosterone (T), Luteinizing Hormone (LH), Follicle-Stimulating-Hormone (FSH), Estrogen (E2), Progesterone (P), anti-Mullerian hormone (AMH) and Sex-Hormone-Binding-Globulin (SHBG)).

### 3.5. Hormone Measurement

Rat specific ELISA kits, (Rat T ELISA kit, Cat No: CSB-E05100r, LH Cat No: CSB-E06869r, FSH Cat No: CSB-E12654r, E2 Cat No: CSB-05110r, P Cat No: CSB-E07282r, AMH Cat No: CSB-E11162r, SHBG Cat No CSB-E08234r, CUSABIO BIOTECH CO., LTD, Japan) were used to measure hormone levels. Intra-assay coefficients of variations for all hormones were less than 10%.

### 3.6. Histological Evaluation

Following anesthetization, adult rats were killed by heart incision; ovaries were removed, placed in 4% paraformaldehyde in phosphate-buffered saline (PBS) at room temperature for 3 days, processed by standard protocols and embedded in paraffin. Sections of the ovary with 4-µm thickness were prepared, deparaffinized by xylene, hydrated by an ethanol series (100%, 90%, 80%, 70% and 50%) and distilled water, and stained using Hematoxylin Harris and Eosin Y (H&E). Sections were mounted and observed by light microscope (100 x magnifications). The number of preantral, antral, preovulatory follicles as well as the number of corpora luteaper ovary were determined by counting 5 representative sections per ovary at least 20 μm apart (2). Follicles and corpora lutea were counted in the ovarian sections with similar size (approximately) between the all groups. To avoid duplicate counting, follicles and corpora lutea were counted by two persons and only follicles with an oocyte visible were counted.

### 3.7. Statistical Analysis

Data are expressed as mean ± SEM. Independent-sample student t test was used to compare the results between the experimental groups and their controls. SPSS 15.0 PC package (SPSS Inc., Chicago, IL) was used for data analysis. P values < 0.05 were considered statistically significant.

## 4. Results

### 4.1. The Number of Female and Male Offspring in Each Group

In control group 1, total number of offspring was 70 (39 females + 31 males), in experimental group 1, was 88 (47 females + 41 males), in control group 2, was 70 (34 female + 36 male) and in experimental group 2, was 72 (44 females + 28 males).

### 4.2. Body weight, Morphological Parameters and Organs Weight

Changes in the body weights are presented in Table 1. There were no significant differences between experimental groups 1 and 2 compared to their controls at any time, except at birth in experimental group 1 compared to its controls. 

Morphological parameters and organs weight in the experimental and control groups are also presented in Table 1. According to our results, AGD in the offspring of both experimental groups was significantly increased compared to their controls at all measurement times (P < 0.001). Clitoris length was significantly increased in the offspring of both experimental groups in comparison to controls (in experimental group 1 P < 0.001 and in experimental group 2 P < 0.05). Nipples were not formed in the offspring of the experimental group 1, whereas in the offspring of the experimental group 2 the number of nipples was unchanged compared to controls. Vaginal length was significantly decreased in the offspring of experimental group 1 compared to controls (P < 0.001), whereas in the offspring of experimental group 2, no significant difference was observed compared to controls (P > 0.05). Male organs including ventral prostate, seminal vesicle and bulbourethral glands were not observed in the female offspring in either of the experimental groups in adulthood.

**Table 1.** Body Weight of Female Offspring of Experimental and Control Groups at Birthday and on Days 15, 30, 45, 60 after birth and in Adulthood and Morphological Parameters and Weight of Organs in Adulthood ^a,b,c^

Parameters	Groups					
	Control 1 (n = 8)	Experimental 1 (n = 9)	P Value	Control 2 (n = 11)	Experimental 2 (n = 9)	P Value
**At** **birth,** **g**	5.59 ± 0.15	6.08 ± 0.15 ^d^	0.04	5. 74 ± 0.11	5.78 ± 0.1	0.83
**On day 15, g**	19.75 ± 1.63	21.13 ± 0.62	0.42	21.72 ± 0.44	24.11 ± 1.87	0.24
**On day 30,** **g**	46.12 ± 4.04	48.11 ± 1.63	0.64	57.27 ± 3.65	54.77 ± 3.68	0.64
**On day 45, g**	87.12 ± 5.8	93 ± 2.9	0.36	87 ± 4.37	80 ± 2.58	0.18
**On day 60, g**	117.87 ± 5.3	126.55 ± 3.39	0.17	125.9 ± 5.35	135 ± 4.87	0.23
**In adulthood, g**	156.25 ± 5.74	171.33 ± 5.01	0.06	162.45 ± 5.69	178.44 ± 9.44	0.14
**Anogenital distance, mm**						
**Day 6**	2.87 ± 0.12	4.44 ± 0.17 ^e^	0	2.63 ± 0.15	4.0 ± 0.28 ^e^	0
**Day 6 30**	7.5 ± 0.18	13.66 ± 0.47 ^e^	0	8.63 ± 0.27	12.88 ± 0.77 ^e^	0
**Day 6 60**	11.12 ± 0.35	19.77 ± 0.27 ^e^	0	10.81 ± 0.26	16.0 ± 0.66 ^e^	0
**AGD in** **adulthood, mm**	11.12 ± 0.35	20.11 ± 0.45 ^e^	0	11.0 ± 0.38	16.22 ± 0.57 ^e^	0
**Clitoris length, mm**	5.25 ± 0.16	8.22 ± 0.52 ^e^	0	4.90 ± 0.36	6.33 ± 0.57 ^d^	0.04
**Nipple count**	12.0 ± 0	0 ^e^	0	12.0 ± 0	12.0 ± 0	0
**Vaginal length, mm**	13.12 ± 0.54	2.77 ± 0.27 ^e^	0	13.72 ± 0.76	13.66 ± 0.23	0.19
**Kidney to ovary distance, mm**	3.62 ± 0.46	5.11 ± 1.18	0.26	5.63 ± 0.57	4.66 ± 0.66	0.28
**Brain weight, g (absolute)**	1.57 ± 0.03	1.65 ± 0.01	0.06	1.63 ± 0.03	1.65 ± 0.04	0.78
**Brain weight (Relative)**	0.0102 ± 0.00038	0.0097 ± 0.00027	0.12	0.0102± 0.00033	0.0094 ± 0.00041	0.14
**Paired adrenal weight, g (absolute)**	0.04 ± 0	0.05 ± 0 ^d^	0.02	0.03 ± 0	0.09 ± 0.05	0.27
**Paired adrenal weight, g (Relative)**	0.0003 ± 0.00002	0.0003 ± 0.00002	0.20	0.0002 ± 0.00002	0.0005 ± 0.00023	0.18
**Paired ovary weight, g (absolute)**	0.10 ± 0	0.20 ± 0.06	0.16	0.11 ± 0	0.12 ± 0.01	0.25
**Paired ovary weight, g (Relative)**	0.0006 ± 0.00004	0.0012 ± 0.00040	0.15	0.0007 ± 0.00003	0.0007 ± 0.00004	0.31

^a^ Values are expressed as mean ± SEM, independent-sample student t test.

^b^ Relative weight is organ weight/body weight.

^c^ Control 1, female offspring of control group 1; Experimental 1, female offspring of experimental group 1; Control 2, female offspring of control group 2; Experimental (2), female offspring of experimental group 2. Adulthood is 90-100 days of age.

^d^ P < 0.05.

^e^ P < 0.001.

### 4.3. Hormone Levels

**Figure 1.**
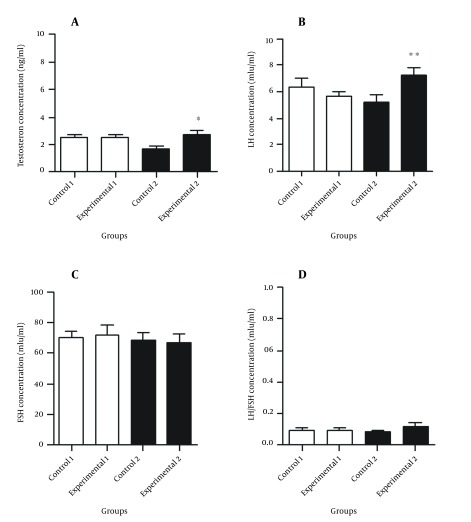
The Hormonal Profiles of Study Groups in Adulthood (90-100 Days of Age) A: Comparison of testosterone concentration, B: Comparison of luteinizing hormone (LH) concentration, C: Comparison of follicle-stimulating-hormone (FSH) concentration, D: Comparison of the ratio of LH/FSH ratio between experimental groups and theirs controls. Values are expressed as mean ± SEM, independent-sample student t test was used. * P < 0.05 and ** P < 0.01.

**Figure 2.**
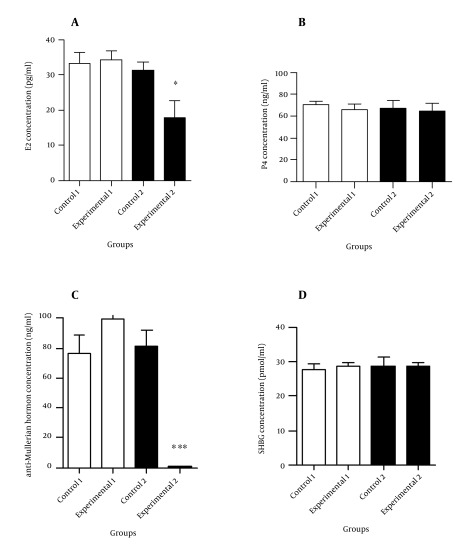
The Hormonal Profiles of Study Groups in Adulthood (90-100 Days of Age) A: Comparison of estrogen concentration, B: Comparison of progesterone concentration, C: Comparison of anti-Mullerian hormone concentration, D: Comparison of sex-hormone-binding-globulin concentration between the experimental groups and controls. Values are expressed as mean ± SEM, independent-sample student t test was used. * P < 0.05 and *** P < 0.001.

Figures 1 and 2 illustrate the hormonal profiles of female offspring of experimental and control groups in adulthood. In experimental group 1, hormonal profiles did not differ compared to its controls, whereas in group 2, levels of testosterone (P ≤ 0.05) and LH (P < 0.01) were significantly increased, and estrogen and anti-Mullerian hormone levels were significantly decreased (P < 0.05 and P < 0.001, respectively) compared to its controls.

### 4.4. Histological Changes of the Ovary

**Figure 3.**
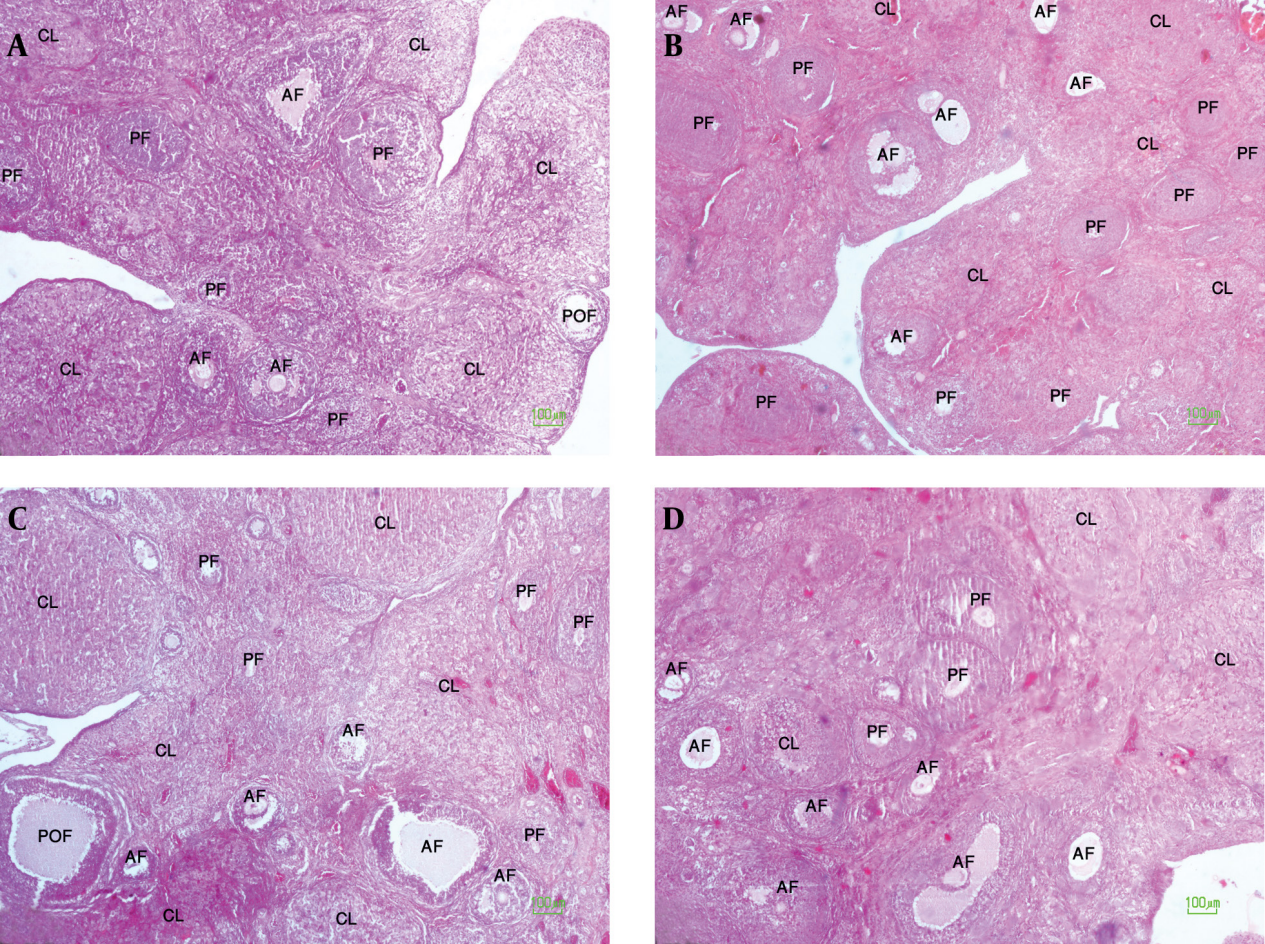
Hematoxylin and Eosin Staining of Rat Ovaries A: ovary tissue in female offspring of control group 1, B: ovary tissue in female offspring of experimental group 1, C: ovary tissue in female offspring of control group 2, D: ovary tissue in female offspring of experimental group 2. PF: Preantral follicle, AF: Antral follicle, POF: Preovulatory follicle, CL: corpus luteum (100 x magnifications).

A significant increase was observed in the number of preantral and antral follicles in the ovaries of offspring of experimental group 1 in comparison to controls (P < 0.001). However, there were no significant differences in the number of preantral and antral follicles of offspring of experimental group 2 in comparison to controls (Table 2 and Figure 3). The number of preantral and antral follicles, preovulatory follicles and corpora lutea per ovary in the female offspring of experimental and control groups are reported in Table 2.

**Table 2.** The Number of Preantral Follicles, Antral Follicles, Preovulatory Follicles and Corpora Lutea per Ovary in Female Offspring of Experimental and Control Groups in Adulthood^a,b,c^

Parameters	Groups					
	Control 1 (n = 5)	Experimental 1 (n = 5)	P Value	Control 2 (n = 5)	Experimental 2 (n = 6)	P Value
**Number of preantral follicles**	4.68± 0.73	8.60 ± 0.91 ^d^	0.01	3.44 ± 0.99	7.40 ± 1.37	0.05
**Number of antral follicles**	5.2 ± 0.53	14.40 ± 2.0 ^e^	0.002	10 ± 2.60	15.60 ± 2.14	0.14
**Number of preovulatory follicles**	0.60 ± 0.35	0.16 ± 0.09	0.27	0.72 ± 0.22	0.53 ± 0.19	0.53
**Number of corpora lutea**	9.28 ± 1.77	10.60 ± 1.87	0.62	10.76 ± 0.99	8.96 ± 1.31	0.32

^a^ Values are expressed as mean ± SEM, independent-sample student t test was used.

^b^ Control 1, female offspring of control group 1; Experimental 1, female offspring of experimental group 1; Control 2, female offspring of control group 2; Experimental 2, female offspring of experimental group 2.

^c^ Adulthood is 90-100 days of age.

^d^ P < 0.05.

^e^ P < 0.01.

## 5. Discussion

The present study showed that exposure of female fetuses to exogenous testosterone during embryonic days 16-19, produced developmental and morphological disorders in reproductive system and androgen-sensitive tissues in female offspring, however its hormonal profiles remained intact. On the other hand, exposure to exogenous testosterone on embryonic day 20, had little effect on the morphology but induced more obvious endocrine disturbances, similar to those observed in PCOS subjects. The novelty of this research was the time of androgen exposure, exposure to exogenous androgen for female fetuses was concurrent with the androgen surge in male fetuses of rats, (an androgen surge is present in male fetuses of rats beginning on embryonic day 16 and lasting until embryonic day 21) (13). This period of female fetus development may be a critical period to androgen exposure. In the present study we aimed to observe polycystic ovary syndrome (PCOS) features in female rats in adulthood by providing prenatal exposure to documented dosages of testosterone at the critical period of fetal development.

Alterations in AGD, nipple, reproductive tract and external genitalia found in our study are in agreement with the findings of previous studies (2, 13-15). Due to the presence of androgen receptors, exposure to androgens before the final development of reproductive tract and androgen-sensitive tissues leads to the male-like morphology in external genital system; some studies reported male-like tissues in their androgenized female animals (13-15) which was not observed in this study; this inconsistency may be explained by differences in the time and androgen dosage duration of exposure and also type of androgen or animal strain.

In the current study, high serum LH concentration in adulthood was observed in female offspring of the experimental group 2, similar to those previously reported in monkey, sheep and mice models (10, 16, 17) Previous studies reported that high frequency of GnRH pulsation in the hypothalamus leads to increased LH secretion from the pituitary gland, but a low frequency of GnRH pulsation leads to FSH secretion (10). Increased LH secretion in our study may be explained through two different mechanisms; first, accelerated GnRH pulse generator activity in the hypothalamus because of prenatal androgen effect; however it remained unclear how prenatal androgen receptor activation may program hyperactivity of GnRH pulse generator in adulthood. In female sheep exposed to androgen during prenatal life, synaptic contacts to GnRH neurons were reduced to lower levels observed in males (18), suggesting that the effects of androgens on GnRH pulsation may be mediated by alterations in synaptic connectivity; another study proposed that androgen receptor activation may create specific alterations in the drive from gamma-aminobutyric acid-releasing neurons to GnRH neurons (10). For the second mechanism, increase in LH may be due to reduction of negative feedback of sex steroids on the LH secretion. As in female monkeys exposed to androgen during early or late gestational period (5, 19, 20) and in women with polycystic ovary syndrome (21, 22) diminished sex steroids negative feedback, leads to increased pulsatile LH secretion in human and animal, however these possible mechanisms were not investigated in our study. 

 In our study an increase in the serum testosterone concentration was observed in female offspring of experimental group 2; this is an indicator of ovarian response to increased LH secretion which acts on theca cells causing elevated testosterone secretion. This finding is supported by an earlier study, where prenatally androgenized rats demonstrated high levels of LH after puberty, which was associated with elevation of testosterone level (2). However, in another study performed on prenatally androgenized mice, level of testosterone did not increase significantly at the age of 5 months despite LH elevation (23). Differences in these results may be explained by differences in the type of animals and their age at the time of examination, considering different results of hormonal profiles in prenatally androgenized animals and also differences in testosterone level at different ages according to previous studies (23, 24). 

Serum FSH concentration in the offspring of experimental group 2 showed no difference compared to controls. A previous study on prenatal androgenized female rats also reported no alterations in the FSH level after puberty (2).

In the present study, a significant decrease was seen in the serum estrogen concentration of offspring of experimental group 2 compared to controls. This reduction may be explained by a decrease in the aromatase enzyme activity. Reduced aromatase activity inhibits the conversion of testosterone to estrogen, leading to low estrogen, but increased levels of testosterone, which may lead to PCOS (25). This hypothesis remains to be examined.

In our study, serum progesterone concentration did not show significant difference in the offspring of experimental group 2 in comparison with its controls, despite a non-significant decreasing trend in the number of corpus luteum. This may be due to the effect of increased LH levels on the corpus luteum, thereby preventing the reduction expected in progesterone levels.

Our findings showed a non-significant decreasing trend in the number of corpora lutea in offspring of experimental group 2, which may represent a decrease in ovulation; following lack of GnRH and LH surges due to prenatal excess of androgen. In female rodents, the preovulation GnRH surge depends on the ability of ovarian E2 to couple a daily neural signal (26-28), a process which appears to be mediated by steroid ability to induce progesterone receptor (Pgr) expression in the anteroventral periventricular (AVPv) nucleus (27, 28). Previous studies have suggested that lack of Pgr expression (29), reduces expression of Pgr in the AVP (27, 30) or the Pgr antagonist (31-35) blocks E2- induced GnRH and LH surges. Studies have shown that prenatal exposure to excess androgen decreases E2-induced Pgr expression in the preoptic area (POA) (2); it has also been shown that prenatal androgen receptors activation leads to permanent refractoriness of the POA to Pgr-inducing actions of E2. Foecking et al. stated that activation of prenatal androgen receptors in female rats can prevent gonadotropin surge release in adulthood (36), as was previously observed in sheep (37-40) and monkeys (41).

Serum anti-Mullerian hormone concentration was significantly reduced in female offspring of experimental group 2, compared to controls. Findings of a studyperformed by Veiga-Lopez et al. on sheep, showed a reduction in expression of anti-Mullerian hormone in the preantral follicles and an increase in its expression in the antral follicles due to prenatal androgen excess (42). 

Lack of changes in the hormone levels of female offspring in the experimental group 1 was in agreement with the results of Foecking et al (36). However, Wu et al. (2) investigated female rats exposed to exogenous androgen during embryonic days 16-19 and observed hormonal changes after puberty (2). Similar to their findings, we observed changes in the number of follicles in female offspring of experimental group 1; this finding may be due to reduced sensitivity of follicles to FSH, which needs to be further investigated. Differences in the time, duration, level of prenatal exposure to androgen, type of androgen and the strain and age of animals at the time of study could explain different results based on different hormonal profiles in prenatally androgenized animals at previous studies (23, 24). 

The main strength of the present study was the time of androgen which was concurrent with production of an androgen surge in male fetuses of rats, (an androgen surge in male fetuses of rats beginning on embryonic day 16 and lasting until embryonic day 21) (13). This period of female fetus development may be a critical period for androgen exposure. In the present study we aimed to observe polycystic ovary syndrome (PCOS) features in female rats in adulthood by providing prenatal exposure to documented dosages of testosterone at the critical period of fetal development. We had some limitations; it is not clear that our observation was due to the differences of exposure time to androgen or the dosage of androgen. However, it has been reported that the effect of androgen on the female reproductive system is highly influenced by the time of exposure (2, 5). As the regards development of genitalia and nervous system of rats begins during fetal life and continues after birth (at birth, the genitalia and nervous system of rats are in the medium-term of differentiation and development), it seems that these systems are more sensitive to androgen in late fetal life. Therefore we think that the observed differences may be mainly influenced by the time of androgen exposure rather than its dosage. However, this needs more investigation.The time of exposure to androgens may have a significant role in the development of certain PCOS characteristics associated with reduction of morphological disorders of the reproductive system. Therefore, avoiding exposure to androgen excess during critical periods of fetal development may prevent or reduce adulthood PCOS manifestations.
